# Annotations

**Published:** 1903-02-28

**Authors:** 


### ANNOTATIONS.

### The Crisis of St. Bartholomew's.

It is confidently stated in well-informed quarters that the Lord Mayor's committee, or a large majority of them, have practically made up their minds to retain St. Bartholomew's Hospital on its present site. If this be true the crisis is already acute, and the life of the patient, as represented by the reputation and position of our greatest medical school, is threatened with danger. This is not the place or the time to show that to perpetuate the hospital of St. Bartholomew's on the enlarged site, as shown in the plan published in the *British Medical Journal* of January 17th, and to leave the existing blocks of wards standing as at present, may result in the medical school and hospital of St. Bartholomew's gradually falling into the second rank of great institutions. The plan in question shows that the superficial area of the site left for wards is so restricted as to cause the number of beds per acre, when the wards are rebuilt on modern lines, to be so large as to be objectionable in a hygienic and modern hospital sense. Further than that, if all the new buildings, which are to cost £350,000 as shown in the plan referred to, are erected, it will never be possible for the Governors without pulling a number of them down to erect upon the site that remains new wards of the highest class. Further, such new wards, having regard to the limited space, must contain very many fewer beds than the hospital possesses at the present time. In these circumstances there would seem to be three alternatives, and three only to select from if St. Bartholomew's Hospital and its great medical school is to be saved from a great disaster. (1) The whole of the existing site must be entirely cleared of buildings, with the exception possibly of the existing medical school buildings. That is to say, a scheme must be prepared at once showing how the hospital and its appurtenances can be modernised and rebuilt according to a carefully thought-out plan which will give the wards the maximum of superficial, air and light space. (2) Arrangements to be made to purchase a further portion of the Christ's Hospital site; or (3) The hospital must be moved to a new and larger site elsewhere. In regard to (1) it may be said that it is made serious on the ground of cost, and that it may entail a further diminution of the existing income of St. Bartholomew's Hospital which it can ill spare, which must entail additions to that income from new sources. Proposal (2) seems to us to be wholly impracticable from a financial point of view, though otherwise feasible. As to (3), failing (2) that is the ideal plan, and it has the further advantage that it will require young and able men of the highest attainments and courage to carry it out. If, however, the committee, or the Governors, or both, with the medical staff, can be induced to take their courage in both hands and adopt this scheme, St. Bartholomew's Hospital will be saved, and its future as the greatest of our medical institutions will be secured for many centuries to come. Plan (3) has the further advantage that it should be capable of being carried out by a great financier on lines which should ultimately result in a large addition to the income and property of St. Bartholomew's Hospital. This is the position to-day in a nutshell, and we shall await the action of the staff and the decision of those immediately concerned with absorbing interest. We fully sympathise with the committee, the Governors, and all concerned in the difficult circumstances of the problem presented. We believe, however, that a wise and adequate solution may be found if sufficient time, thought and brains be brought to bear upon it. Everybody who has the capacity and power to help should willingly lend a hand, for St. Bartholomew's Hospital is deservedly regarded as probably our greatest medical charity and the premier medical school of the nation.

### The Health Aspects of London Locomotion.

The appointment of a Royal Commission to inquire into the means of locomotion and transport in London is a matter of great importance to all dwellers and workers in the metropolis, for the inquiry which is about to be made may not impossibly determine the character of the future London, and will almost certainly have a considerable influence in deciding the directions in which it shall grow. It may savour somewhat of a paradox to suggest that any large increase in the facilities for locomotion between the centre and the periphery of this great city, unless effected with great care and with more forethought than has hitherto been given to it, may prove injurious to the health of the workers, but it is none the less true. To those who live and work within such a province of bricks and mortar as London has become, and under such a pall of smoke and grime as that in which it lies buried there is something peculiarly delightful in the idea of a suburban home. Workers, however, soon find out that the hour's journey at each end of the day's work is no small addition to the day's labour, and that the boasted healthiness of the "growing" suburb is somewhat of a myth while its dulness is undoubted, and, unless great care is exercised in ensuring the healthiness of the means of transit provided, any large increase in facilities for locomotion will almost certainly condemn the workers, who will be practically compelled to make use of them, to a twice-a-day exposure to many risks to health. In an ideal city factories and workshops would stretch far out into the suburbs, and all those engaged in manual labour would work within an easy walk of their homes The stifling journey in an overcrowded "tube," carrying a double row of extra passengers all down the gangway, is no substitute for the walk and the blow of fresh air which every worker ought to have between his workshop and his home, and unless the Royal Commission bears in mind the dangers to health involved in some modern means of locomotion, anything which by way of increased cheapness or convenience practically ties down large masses of the community to use them may in the end have very serious consequences. The Royal Commission should regard the problem from a sanitary, quite as much as from a geographical, mechanical, or even financial point of view.

